# Genomic and functional characterization of *six* novel phages targeting multidrug*-*resistant *Escherichia coli*: prom*isi*ng candidates for clinical phage therapy

**DOI:** 10.3389/fmicb.2026.1921205

**Published:** 2026-09-10

**Authors:** Lingling Xiong, Xiaolian Shen, Yingying Xiang, Jingting Sun, Ting Wang, Qian Zhou, Kehui Xiong, Wenxue Wang, Jiawei Geng, Xiuling Ji

**Affiliations:** 1 Faculty of Life science and Technology & Medical School, Kunming University of Science and Technology, Kunming, China; 2 Department of Stomatology, Yan’an Hospital Affiliated to Kunming Medical University, Kunming, China; 3 Department of Infectious Disease and Hepatic Disease, The First People’s Hospital of Yunnan Province, Kunming University of Science and Technology Affiliated Hospital, Kunming, China

**Keywords:** antimicrobial resistance, bacteriophage, biofilm, *Escherichia coli*, phage therapy

## Abstract

Antimicrobial resistance in multidrug-resistant *Escherichia coli* has severely compromised the efficacy of conventional antibiotics, particularly in hospital-acquired infections. Bacteriophage therapy offers a targeted approach, yet the rapid emergence of phage-resistant mutants during monotherapy limits its durability. However, phage cocktail therapy can overcome this issue. In this study, we isolated and characterized six novel lytic bacteriophages from hospital wastewater that specifically infect MDR *E. coli* clinical isolates. All six phages exhibited strictly lytic lifestyles, and genomic analysis confirmed the absence of lysogeny, virulence, or antibiotic-resistance genes. They are promising candidates for phage cocktail therapy. Stability assays demonstrated that the phages maintained activity under various temperature and pH conditions. Adsorption kinetics showed rapid binding to host cells, and one-step growth curves indicated efficient lytic replication with short latent periods and high burst sizes. Moreover, the phages consistently reached high titers (10^10^–10^12^ PFUs/mL) and demonstrated potent activity against preformed biofilms of MDR *E. coli* strains. Phylogenetic analyses placed these phages within distinct clusters, supporting their genetic diversity. Together, these findings expand current understanding of the biology and diversity of *E. coli*-targeting phages and provide a robust foundation for the rational design of phage cocktails aimed at overcoming persistent MDR *E. coli* infections.

## 1 Introduction

Antimicrobial resistance (AMR) has emerged as one of the most critical global public health threats of the 21st century, significantly compromising the effectiveness of conventional antibiotic therapies (Marino et al., 2025). Pathogens such as *Enterococcus faecium*, *Staphylococcus aureus*, *Klebsiella pneumoniae*, *Acinetobacter baumannii*, *Pseudomonas aeruginosa*, and Enterobacteriaceae are collectively referred to as ESKAPE pathogens (Miller and Arias, 2024). The rapid increase in multidrug-resistant (MDR) bacterial pathogens has led to the rise in morbidity, mortality, and healthcare costs worldwide, highlighting the urgent need for alternative antimicrobial strategies (Chinemerem Nwobodo et al., 2022).

Among antimicrobial-resistant pathogens, *Escherichia coli* represents a particularly important clinical and public health concern because of its dual role as a common commensal organism and an opportunistic pathogen (Miller and Arias, 2024; Palacios-Baena et al., 2021; Tacconelli et al., 2018; Yip et al., 2023). Among MDR pathogens, *E. coli* poses a considerable clinical challenge due to its dual role as both a commensal organism and an opportunistic pathogen (Aljohni et al., 2025; Zegene et al., 2025). MDR *E. coli* strains, particularly those producing extended-spectrum β-lactamases (ESBLs), are associated with a diverse array of infections, including urinary tract infections, bloodstream infections, neonatal meningitis, pneumonia, and gastrointestinal diseases (Aljohni et al., 2025; Manges et al., 2019; Tenaillon et al., 2010; Zegene et al., 2025; Zhou et al., 2015). The rising prevalence of antibiotic-resistant *E. coli* has severely limited treatment options, presenting a persistent challenge to global healthcare systems (Danaei et al., 2023). These challenges highlight the need for alternative antimicrobial approaches that can effectively target MDR *E. coli* while minimizing the collateral disruption of the host microbiota.

In response to the growing challenge posed by MDR *E. coli*, bacteriophage therapy has emerged as a promising alternative antimicrobial strategy (Galtier et al., 2017; Jun et al., 2014; Kim et al., 2025). Bacteriophages can selectively infect and lyse bacterial cells, offering high host specificity, self-amplifying antibacterial activity at sites of infection, and comparatively limited disruption of the surrounding microbiota (Aranaga et al., 2022; Mafe and Büsselberg, 2025; Strathdee et al., 2023). Their ability to specifically target MDR *E. coli*, including within complex microbial communities, further supports their potential application in precision antimicrobial therapy (Larsson et al., 2026; Shuwen and Kefeng, 2022; Zalewska-Piątek and Piątek, 2020). Previous studies have demonstrated therapeutic benefits of phages against *E. coli* infections in both experimental and clinical settings. For example, prophylactic phage administration reduced intestinal colonization and attenuated colitis following enteroinvasive *E. coli* challenge in a mouse model (Galtier et al., 2017). In addition, successful treatment of recurrent MDR *E. coli* infection in a patient with chronic bacterial prostatitis has highlighted the potential of phage therapy when conventional antibiotics fail (Johri et al., 2023). Several other studies have provided further evidence supporting the therapeutic potential of phages against MDR bacterial infections (Dedrick et al., 2023; Federici et al., 2022; Mafe and Büsselberg, 2025; Olawade et al., 2024; Weiner et al., 2025).

However, the clinical application of phage therapy is frequently constrained by the rapid emergence of bacterial resistance. Experimental and clinical studies have demonstrated that phage-resistant bacterial subpopulations can arise within hours to days of exposure to individual phages, potentially resulting in bacterial regrowth and treatment failure (Aslam et al., 2024; Cardoso et al., 2025; Fujiki et al., 2025). In both *E. coli* and *P. aeruginosa* infection models, resistance to single phages has been associated with reduced therapeutic efficacy, while clinical cases have reported transient bacterial clearance followed by relapse associated with the emergence of phage-resistant variants (Blasco et al., 2023; Pirnay et al., 2024). These resistance dynamics limit the durability of monovalent phage therapy and highlight the need for strategies that can reduce treatment escape while maintaining effective bacterial killing.

To overcome the limitations of phage monotherapy, phage cocktails comprising multiple phages with distinct receptor specificities and infection characteristics have been proposed as a promising strategy (Kaneko et al., 2026; Kim et al., 2025). By targeting bacteria through different infection mechanisms or cellular receptors, phage cocktails may broaden antibacterial coverage, reduce the probability of resistance emergence, and potentially improve bactericidal efficacy. This strategy is particularly relevant for genetically diverse MDR *E. coli* populations and is consistent with the concept of precision antimicrobial therapy. However, the development of effective phage cocktails requires the discovery and characterization of diverse phages with complementary biological and genomic properties. Clinically relevant environments, including hospital wastewater, represent potentially valuable reservoirs for isolating phages that are adapted to MDR bacterial populations. Nevertheless, the diversity and detailed characterization of *E. coli* infecting phages from such environments remain insufficiently explored.

In this study, we isolated and characterized six novel bacteriophages targeting MDR *E. coli* from hospital wastewater. All six isolates were strictly lytic, and their biological characteristics were systematically evaluated. We investigated their host ranges, environmental stability, adsorption efficiency, and lytic activity, and performed genomic and phylogenetic analyses to assess their genetic features and evolutionary relationships. Collectively, this study provides a foundation for evaluating the complementary properties of these phages and their potential utility in the future development of phage cocktails against MDR *E. coli*.

### 2 Materials and methods

#### 2.1 Bacterial strains and culture condition

The clinical multidrug-resistant *E. coli* strains were isolated from the fecal samples from patients in the Department of Infectious Diseases and Hepatic Disease at First People’s Hospital of Yunnan Province. The isolates had been previously recovered from fecal clinical specimens by the hospital and were subsequently provided to our laboratory for microbiological research. No patient-identifying information or clinical data were made available to the authors. These strains were utilized as the host bacterium for phage isolation and characterization, and were routinely cultured in brain heart infusion (BHI) medium at 37°C.

For molecular identification, each of the six clinical isolates was subjected to 16S rRNA gene amplification and sequencing using the universal bacterial primers 27F and 1492R (Han, 2006). PCR amplification was performed for 30 cycles under the following conditions: initial denaturation at 94°C for 2 min, followed by denaturation at 94°C for 30 s, annealing at 52°C for 30 s, and extension at 72°C for 40 s, with a final extension at 72°C for 10 min. The resulting PCR products were purified and sequenced. The obtained 16S rRNA sequences were compared with the NCBI non-redundant (Nr) database using BLASTn to confirm the species-level identification of each isolate (Camacho et al., 2009).

#### 2.2 Antibiotic susceptibility testing of bacterial strains

We employed the 2025 standards of the Clinical and Laboratory Standards Institute (CLSI) and used the Kirby-Bauer disc diffusion method to determine the inhibition zone diameters of *E. coli* against common antimicrobial agents for phage lysis spectrum analysis (Clinical and Laboratory Standards Institute [CLSI], 2025). The antibiotics used include Tetracycline (TE, 30 μg), Doxycycline (DX, 30 μg), Minocycline (MI, 30 μg), Lincomycin (LC, 2 μg), Clindamycin (CC, 2 μg), Levofloxacin (LVX, 5 μg), Metronidazole (MTZ, 5 μg), Gentamicin (GM, 10 μg), Ertapenem (ETP, 10 μg), Meropenem (MRP, 10 μg), Imipenem (IMI, 10 μg), and Ceftazidime (CAZ, 30 μg). In this study, MDR was defined as acquired non-susceptibility to at least one agent in three or more antimicrobial categories (Magiorakos et al., 2012). Resistance to imipenem, meropenem, or ertapenem was defined as Carbapenem-Resistant *E. coli* (CREC) strains (Diab et al., 2026).

#### 2.3 Phage isolation and purification

Phage isolation: *E*. *coli* strains B21, B49, B62, B70, B119, and BE17 were used as bacterial hosts for phage isolation. Wastewater samples collected from Yan’an Hospital, Yunnan, China, were used as environmental sources for phage isolation. Briefly, wastewater samples were mixed with BHI broth and incubated statically at room temperature for 3–4 days to allow the amplification and release of naturally occurring phage particles in the wastewater samples. Following incubation, samples were centrifuged at 10,000 *g* for 5 min, and the supernatants were filtered through 0.22 μm Millipore membranes to remove bacterial cells and debris (Lone et al., 2024). The presence of lytic phages was subsequently screened using a spot test assay against the corresponding MDR-*E. coli* hosts. Briefly, filtered supernatants were spotted onto double-layer agar plates containing the corresponding host bacteria. The appearance of clear lytic zones indicated the presence of phages with detectable lytic activity. Samples showing positive lysis were subsequently subjected to plaque assays for phage isolation and purification (Lone et al., 2024).

Phage purification and preparation of phage stocks: Individual phages were purified through multiple rounds of single plaque isolation using the double-layer agar plate method until uniform and well-defined plaques were obtained. Purified phages were propagated on their corresponding host strains to generate high-titer lysates. For preparation of purified phage stocks, phage lysates were concentrated by polyethylene glycol (PEG) precipitation, followed by chloroform extraction and ultrafiltration to remove residual bacterial components and impurities. The purified phage preparations were suspended in SM buffer, stored at 4°C, and used for subsequent biological characterization and genomic analyses (Ács et al., 2020; Shaaban et al., 2026).

#### 2.4 Host range assay

The host range of the isolated phages was evaluated using a spot assay as previously described with minor modifications (Li et al., 2026). Briefly, overnight bacterial cultures of different test strains were spread onto BHI agar plates to form bacterial lawns. After the plates were dried, 5 μL of serially diluted phage lysates were spotted onto the bacterial lawns. The plates were incubated at 37°C for 12 h, and phage infectivity was determined based on the formation of clear lytic zones. The presence of clear plaques or lysis zones was considered indicative of successful phage infection.

#### 2.5 Transmission electron microscopy

Phage morphology was examined using transmission electron microscopy. Purified phage stocks prepared as described above were used for transmission electron microscopy (TEM) analysis. A drop of phage suspension (approximately 10^10^ PFU/mL) was applied onto carbon-coated copper grids and allowed to adsorb. The grids were negatively stained with 2% uranyl acetate and examined using an HT7800 transmission electron microscope operated at 80 kV (Ohi et al., 2004).

#### 2.6 Multiplicity of infection, adsorption assay, and one-step growth curve

The optimal multiplicity of infection (MOI) was determined as previously described (Zhang et al., 2023), with minor modifications. Briefly, phage lysates were added to exponentially growing *E. coli* cultures at MOIs of 100, 10, 1, 0.1, and 0.01, respectively. The mixtures were incubated at 37°C with shaking at 180 rpm for 4 h. Following incubation, samples were centrifuged at 8,000 *g* for 10 min at 4°C, and the supernatants were filtered through a 0.22 μm sterile membrane. Phage titers were then determined using the double-layer agar plaque assay. The MOI yielding the highest phage titer was defined as the optimal MOI. All experiments were performed in triplicate.

The adsorption kinetics of phages were evaluated as previously described (Chen et al., 2025), with minor modifications. Phages were mixed with exponentially growing host bacteria at the optimal MOI and incubated at 37°C. At 5-min intervals up to 30 min, 1 mL samples were collected. Each sample was promptly centrifuged at 12,000 *g* for 2 min at 4°C to separate free phages from bacterial cells. The supernatant was then collected, and phage titers were determined using the double-layer agar method. The adsorption rate was calculated using the following equation:


$$\mathrm{Free}\mathrm{phage}\left(\%\right)=N/{\text{N}}_{\text{0}}\times 100\%$$

where N_0_ represents the initial phage titer at time 0, and N represents the phage titer at each subsequent time point (5, 10, 15, 20, 25, and 30 min).

One-step growth curve: The one-step growth curve was determined as previously described (Pang et al., 2026). Phages were mixed with exponentially growing host bacteria at the optimal MOI and incubated at room temperature for 10 min to allow adsorption. The mixture was then centrifuged at 10,000 *g* for 1 min at 4°C. The pellet was washed three times with BHI medium to remove unabsorbed phages and resuspended in 30 mL of fresh BHI medium. The suspension was incubated at 30°C with shaking at 180 rpm. At 5 min intervals up to 120 min, 1 mL samples were collected and centrifuged at 10,000 *g* for 2 min at 4°C. Phage titers in the supernatant were determined using the double-layer agar plaque assay. All experiments were performed in triplicate.

#### 2.7 Host killing curve assays

The antibacterial activity of phages was evaluated by monitoring bacterial growth inhibition at different multiplicities of infection (MOIs: 100, 10, 1, 0.1, 0.01, and 0.001) (Essam et al., 2025). Exponentially growing *E. coli* cultures were used as the host system. For each condition, 100 μL of bacterial suspension was mixed with 100 μL of phage suspension in a 96-well microtiter plate to achieve the desired MOI. The plates were incubated at 37°C with continuous shaking at 180 rpm for 7 h. During incubation, bacterial growth was monitored by measuring optical density at OD_600_ at regular intervals (30–60 min). A bacterial culture without phage addition served as the positive growth control, while sterile BHI medium was used as a blank control. All experiments were performed in triplicate.

#### 2.8 Bacteriophage lytic kinetics assay

The lytic kinetics and bactericidal activity of phages were evaluated at the optimal MOI determined in previous assays (Essam et al., 2025; Konopacki et al., 2020). Briefly, host bacteria were cultured in BHI broth at 37°C with shaking at 180 rpm, and then subcultured at 1% (v/v) into fresh BHI medium until reaching the mid-logarithmic growth phase (OD_600_ about 0.5). At this stage, phage suspensions were added at the optimal MOI. Cultures were further incubated under the same conditions, and bacterial growth was monitored by measuring OD_600_ at 30–60 min intervals for up to 9 h. Uninfected bacterial cultures grown under identical conditions served as controls. All experiments were performed in triplicate. Bacterial growth curves were generated by plotting OD_600_ values against incubation time, and phage-mediated inhibition was assessed by comparing treated groups with the corresponding uninfected controls.

#### 2.9 Thermal and pH stability of bacteriophages

The thermal and pH stability of bacteriophages were evaluated as previously described (Sun et al., 2024), with minor modifications. For thermal stability, equal aliquots (0.5 mL) of phage lysates were incubated at different temperatures (4, 15, 28, 37, 45, 55, 65, and 70°C) for 1 h. For pH stability, 100 μL of phage suspension was mixed with 900 μL of PBS adjusted to different pH values ranging from 3 to 12, and incubated at 37°C for 1 h. After treatment, samples were serially diluted, and surviving phage titers were determined using the double-layer agar plaque assay. Purified phage stocks had titers of 10^10^–10^12^ PFU/mL (Table 1). All experiments were performed in triplicate.

**TABLE 1:** Morphology and genomic features of the isolated phages.

Phage	P21	P49	P62	P70	P119	PE17
Morphological type	Myovirus-like morphology	Myovirus-like morphology	Siphovirus-like morphology	Myovirus-like morphology	Myovirus-like morphology	Myovirus-like morphology
Phage stock titer (PFUs/mL)	1∼4 × 10 ^11^	1∼4 × 10^10^	1∼4 × 10^10^	1∼4 × 10^10^	1∼4 × 10^11^	1∼4 × 10^12^
head diameter (nm)	33.2 ± 2.2	44.6 ± 3.1	34.6 ± 0.4	58 ± 3.6	32.6 ± 1.95	39.8 ± 2.8
tail length (nm)	99.4 ± 2.8	69.2 ± 2.3	129.9 ± 3.1	79.9 ± 2.5	61.9 ± 2.6	83.2 ± 2.4
Genome size (bp)	167,381	171,839	93,752	171,456	27,851	114,216
GC%	38.14	40.16	45	41	40	38.57
Number of predicted genes	266	294	153	296	35	195
Hypothetical proteins	166	205	149	207	16	137
Genes with predicted function	100	89	4	89	19	58
Lifestyle	Virulent	Virulent	Virulent	Virulent	Virulent	Virulent
tRNA	10	2	1	2	0	2
Antibiotic resistance genes	None	None	None	None	None	None
Virulence genes	None	None	None	None	None	None
Phylogenetic classification	Family: *Straboviridae*; Subfamily: *Tevenvirinae*; Genus: *Tequatrovirus*	Family: *Straboviridae*; Subfamily: *Tevenvirinae*; Genus: *Gaprivervirus*	Class: *Caudoviricetes*; *Caudoviricetes* incertae sedis	Family: *Straboviridae*; Subfamily: *Tevenvirinae*; Genus: *Gaprivervirus*	Family: *Straboviridae*; Subfamily: *Tevenvirinae*; Genus: *Gaprivervirus*	Family: *Straboviridae*; Subfamily: *Tevenvirinae*; Genus: *Gaprivervirus*

#### 2.10 Phage genome sequencing, annotation, and bioinformatic analysis

Phage genomic DNA sequencing was performed by MAGIGENE (China) using the Illumina NovaSeq platform. Viral DNA was extracted using the Norgen Biotek DNA purification kit (Thorold, Canada) according to the manufacturer’s instructions. DNA purity and concentration were assessed using a NanoPhotometer and Qubit fluorometer. Sequencing libraries were constructed using the NEBNext^®^ DNA Library Prep Kit. Raw sequencing data were processed using SOAPnuke (Chen et al., 2018) for quality control, followed by read trimming with Trimmomatic (Bolger et al., 2014). High-quality reads were assembled de novo using MEGAHIT (Li et al., 2015), and assembly quality was evaluated using BWA (Li and Durbin, 2010). Viral contigs were identified by comparison against the CheckV database using CheckV (Nayfach et al., 2021), and genome completeness and quality were further assessed. Genome circularization was assessed based on terminal overlaps between contig ends. Contigs with terminal overlaps longer than 50 bp and sequence identity higher than 94% were circularized during genome assembly analysis.

Gene prediction was performed using Prokka (Seemann, 2014), and predicted open reading frames (ORFs) were annotated by alignment against the UniProtKB/Swiss-Prot viral protein database (ViralZone,),^1^ KEGG,^2^ and the COG database,^3^ allowing functional characterization of viral proteins. Putative phage genomes were further screened using VirSorter2 (Roux et al., 2015), which classified viral contigs based on viral hallmark genes, similarity to known viral sequences, and host-related features. The resulting annotations were converted into a format compatible with DRAM-v (Shaffer et al., 2020), which was used to identify and functionally classify auxiliary metabolic genes (AMGs). AMG types and metabolic categories were subsequently summarized.

Taxonomic classification was determined based on NCBI BLASTn results. Virulence-associated genes, antibiotic resistance genes, lifestyle-related genes, and anti-CRISPR elements were identified using the Virulence Factor Database (VFDB),^4^ the Comprehensive Antibiotic Resistance Database (CARD),^5^ and tRNAscan-SE 2.0,^6^ respectively. Phage lifestyle prediction was performed using PhaTYP.^7^

Genome visualization was performed using Proksee (Grant et al., 2023) based on annotated genomic features and functional classification results. For phylogenetic analysis, the top 50 BLASTn hits were retrieved, and maximum-likelihood phylogenetic trees were constructed from Newick-formatted alignments and visualized using MEGA X (Kumar et al., 2018). Phylogenetic analyses were further performed based on amino acid sequences of the major capsid protein (Mcp), terminase large subunit (TerL), and endolysin. The top 100 BLASTp hits for each marker gene were retrieved for constructing the phylogenetic trees. Average nucleotide identity (ANI) analysis was conducted using OrthoANI (Lee et al., 2016), comparing the phage genomes obtained in this study with representative genomes from the NCBI database. A total of the top nine reference phage genomes were included, and ANI heatmaps were generated to evaluate genomic relatedness. Finally, proteome-based phylogenetic relationships among closely related phages were inferred using ViPTree v3.3 (Nishimura et al., 2017), providing whole-genome-based evolutionary insights into viral taxonomy and relatedness.

#### 2.11 Biofilm removal assay

Biofilm formation and removal were evaluated using a crystal violet staining assay (Huang et al., 2025a). Briefly, logarithmic-phase bacterial cultures were diluted in fresh BHI medium to a concentration of 10^7^ CFU/mL. Then, 100 μL of the diluted culture was added to a 96-well sterile plate, and then incubated at 37°C without rotation for 24 h. Phage preparations at optimal MOI were then added to the preformed biofilms, followed by incubation at 37°C for an additional 6 and 12 h. The bacterial culture was incubated in BHI medium as the control group, while the experimental group was co-cultured with different phages. After biofilm maturation, the planktonic phase was removed, and wells were washed three times with PBS. After treatment, biofilm biomass was quantified using the crystal violet staining method. The remaining biofilms were stained with 0.1% (w/v) crystal violet solution (150 μL per well) for 10–15 min at room temperature. Excess dye was removed by rinsing with distilled water until the blank control wells were visually colorless, and the plates were air-dried. The bound crystal violet was then solubilized using 95% ethanol (150 μL per well), and the absorbance was measured at 595 nm using a microplate reader. All experiments were performed in triplicate.

#### 2.12 Statistical analysis

All experiments were performed using GraphPad Prism 8. Statistical significance was determined using Student’s *t*-test or one-way ANOVA for quantitative measurements. Differences were considered statistically significant at *p*-values of < 0.05 (*), < 0.01 (**), or < 0.001 (***).

### 3 Results

#### 3.1 Phenotypic antimicrobial susceptibility testing (AST) of *E. coli* isolates

The *E. coli* strains B21, B49, B62, B70, B119, and BE17 exhibited resistance to most of the tested antibiotics, including β-lactam antibiotics, Tetracyclines, Lincosamides, Fluoroquinolones, Nitroimidazoles, and Aminoglycosides (Supplementary Table S1). Therefore, B21, B49, B62, B70, B119, and BE17 are classified as multidrug-resistant strains. Among these, B49, B119, and B70 exhibited resistance to imipenem, meropenem or ertapenem, categorizing them as CREC strains (Supplementary Table S1).

#### 3.2 Phage isolation and morphology

**FIGURE 1:**
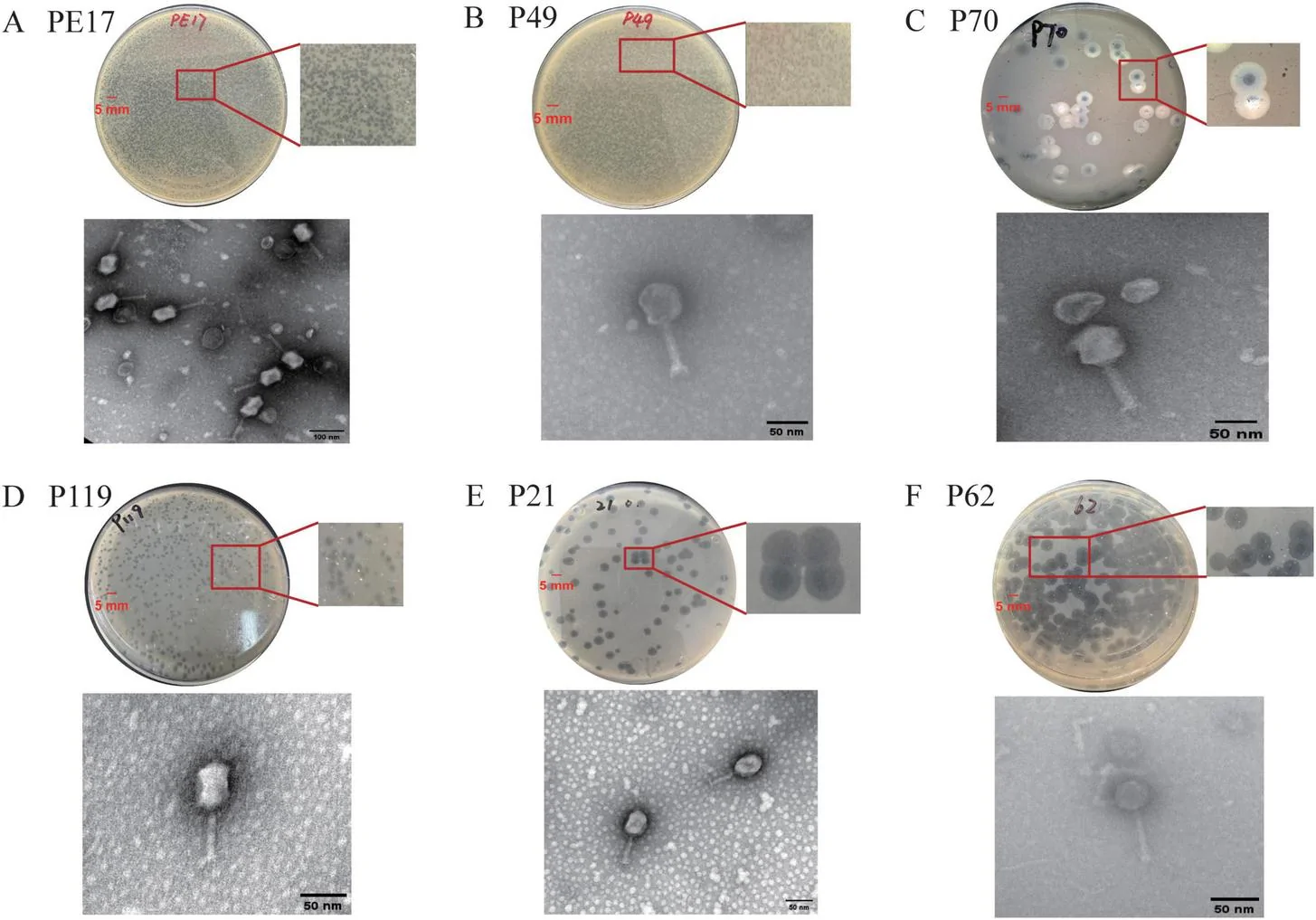
Morphological observation of the six phages. **(A)** Plaques formed by phage PE17 on a bacterial lawn and TEM image of phage PE17. **(B)** Phage P49. **(C)** Phage P70. **(D)** Phage P119. **(E)** Phage P21. **(F)** Phage P62.

The six lytic *E. coli* phages P21, P49, P62, P70, P119, and PE17 were isolated from sewage collected at the hospital using a double-agar layer assay. The double-layer plaque assays produced well-defined, clear lysis zones/plaques, with clear circular plaques under the tested conditions. Purified phage stocks obtained after propagation at 37°C reached titers ranging from 1∼4 × 10^10^ to 1∼4 × 10^12^ PFUs/mL (Table 1), with individual titers of P21, P49, P62, P70, P119, and PE17 subsequently used for phenotypic characterization and genome-based analyses. TEM revealed that all six isolated phages exhibited typical tailed phage morphologies, suggesting that they belong to the morphological group commonly observed among members of the class *Caudoviricetes* (Figure 1). The morphological characteristics, including head diameter and tail length, are summarized in Table 1. P21, P49, P62, P70, P119, and PE17 exhibited distinct capsid and tail dimensions.

#### 3.3 Host range determination

The host range profiles of the six isolated phages against different MDR *E. coli* strains are summarized in Supplementary Table S2. Phages PE17, P49, P62, and P70 exhibited strict host specificity, exclusively lysing their original hosts. In contrast, Phage P119 showed a broad host range by lysing five out of six *E. coli* strains, while Phage P21 lysed three. Notably, none of the tested phages infected *Klebsiella pneumoniae and Staphylococcus aureus*, confirming their high specificity for *E. coli*.

#### 3.4 One-step growth analysis and multiplicity of infection

**FIGURE 2:**
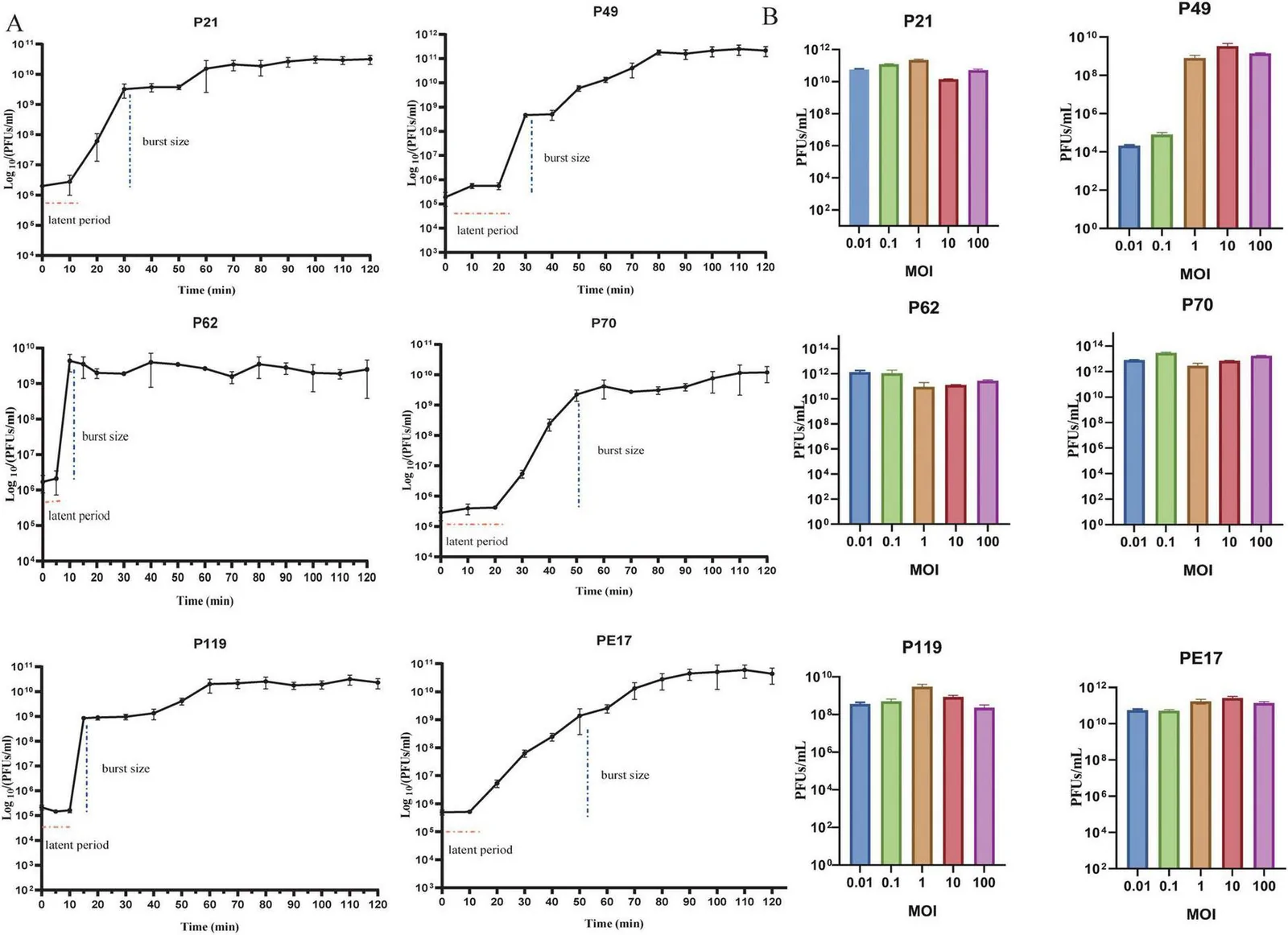
One-step growth curves and multiplicity of infection (MOI) analysis of six bacteriophages. **(A)** One-step growth curves of six bacteriophages, showing the latent period (red), burst period (blue). **(B)** MOI analysis of the six bacteriophages under different multiplicity of infection conditions, used to evaluate their optimal infection efficiency.

As shown in Figure 2, all six bacteriophages (P21, P49, P62, P70, P119, and PE17) were able to effectively infect and propagate within their host bacteria, and differences in their growth kinetics and optimal MOI were observed. The one-step growth curve analysis revealed that all phages exhibited typical infection dynamics, including a latent period, a rise period, and a plateau phase. Specifically, P21, P49, P62, P70, P119, and PE17 showed a gradual increase in phage titer over time, followed by stabilization at later stages, indicating sustained replication capacity. However, their one-step growth kinetics differed substantially. This allowed the transition from the initial infection stage to phage release to be more accurately resolved (Figure 2A). Based on the one-step growth curves, the latent periods and burst sizes were estimated as follows. P21 exhibited a latent period of approximately 10 min and a burst size of approximately 40 PFU/infected cell. P49 showed a latent period of approximately 20 min and a burst size of approximately 25 PFU/infected cell. P62 exhibited a latent period of approximately 5 min and a burst size of approximately 52 PFU/infected cell. P70 showed a latent period of approximately 20 min and a burst size of approximately 30 PFU/infected cell. P119 exhibited a latent period of approximately 10 min and a burst size of approximately 32 PFU/infected cell, whereas PE17 showed a latent period of approximately 10 min and the largest burst size of approximately 260 PFU/infected cell. Overall, the results revealed considerable differences in infection kinetics among the six phages, with latent periods ranging from approximately 5–20 min and burst sizes ranging from approximately 25–260 PFU/infected cell.

The MOI optimization results further demonstrated that the optimal infection conditions varied among the phages. P21 and P119 achieved relatively high titers at an MOI of 1, whereas P49 and PE17 performed better at an MOI of 10. Phage P62 and P70 reached their highest titer at a MOI of 0.1, indicating that 0.1 is its optimal MOI (Figure 2B). Taken together, these results indicate that all six bacteriophages possess strong infectivity and replication ability; however, their optimal infection parameters differ. These findings provide a basis for selecting appropriate inoculation ratios in subsequent applications.

#### 3.5 Bacteriophage adsorption rate

**FIGURE 3:**
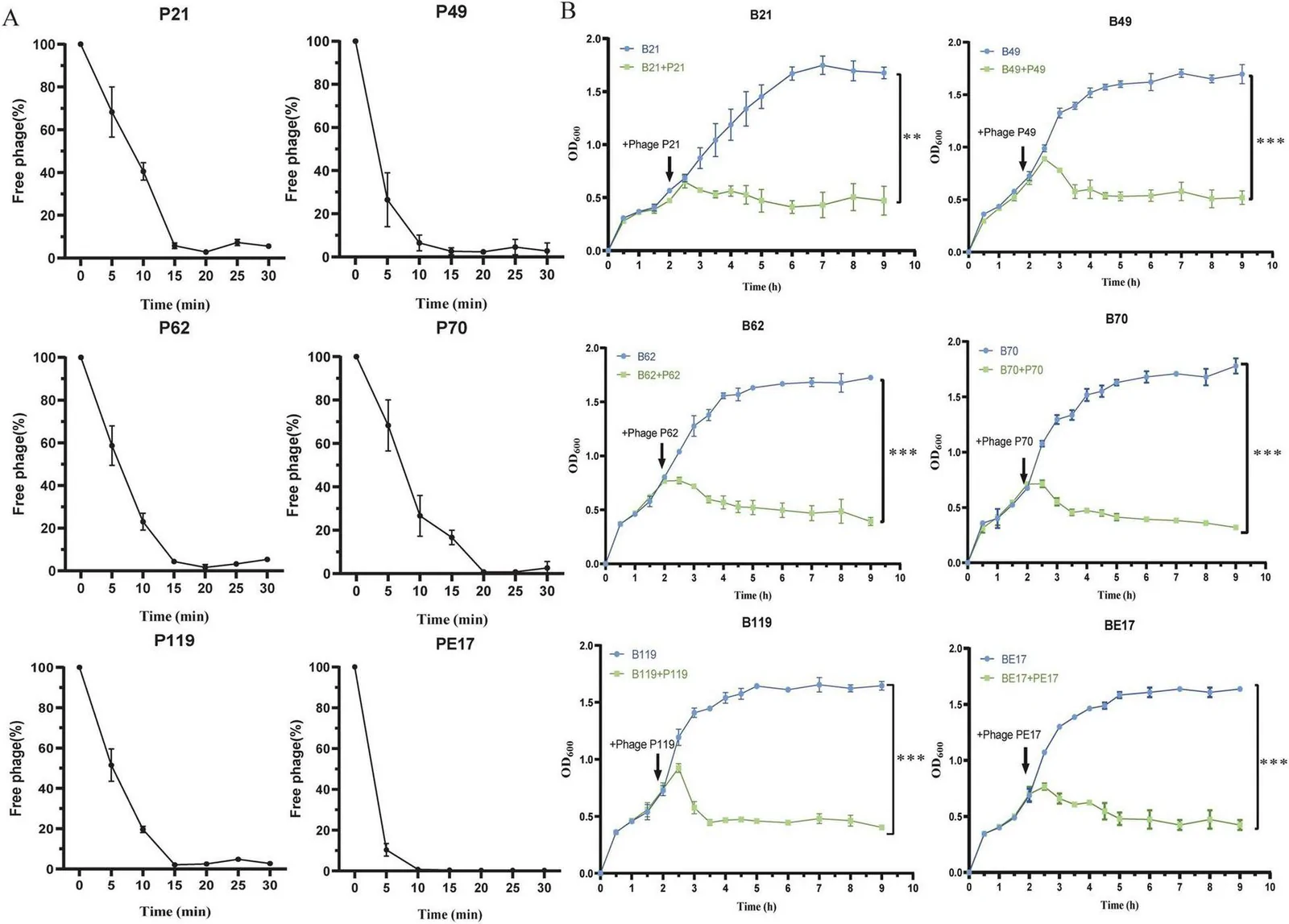
Adsorption curves and host killing curves of the six phages. **(A)** Adsorption curves to their host strains. **(B)** Lytic kinetics of the six phages against *E. coli*. Values are expressed as means ± the SD. The * indicates statistically significant differences, with *p < 0.05; **p < 0.01, ***p < 0.001 (one-way ANOVA).

To characterize the adsorption kinetics of the six phages to their respective host cells, adsorption rates were measured over time. The adsorption efficiency was assessed by detecting the residual phage content in the mixture after adsorption. The results showed that when phage P21 was adsorbed for 15 min, the adsorption rate of 94.19%. When phage P49 was adsorbed for 10 min, the residual phage content was the lowest, with an adsorption rate of 93.53%. When phage P62 was adsorbed for 15 min, with an adsorption rate of 95.57%. When phage P70 was adsorbed for 20 min, with an adsorption rate of 99.24%. When phage P119 was adsorbed for 15 min, with an adsorption rate of 97.88%. When phage PE17 was adsorbed for 5 min, with an adsorption rate of 89.62% (Figure 3A). These six phages exhibited high adsorption rates toward their host cells.

#### 3.6 Host killing curve of phages

To characterize the abilities of phages against their *E. coli* host, the host killing curves were generated for six phages. The host killing curves of the six phages were derived at MOIs ranging from 0.001 to 100. The most significant inhibition of bacterial growth, as indicated by no increase in OD_600_ nm, was observed at different MOI for the 7 h infection, respectively (Supplementary Figure S1).

To evaluate the lytic efficiency of the six phages, we monitored the OD_600_ of *E. coli* over 9 h. In the absence of phage (control group), B21 exhibited robust growth, reaching the stationary phase with an OD_600_ of approximately 1.7 by the end of the experiment. In contrast, upon addition of phage P21 at the mid-logarithmic phase (OD_600_ ≈ 0.6, indicated by the arrow), the bacterial growth was immediately arrested. The OD_600_ of the infected culture gradually declined over the remaining 7 h, indicating successful bacterial clearance. Statistical analysis revealed a highly significant difference in bacterial density between the control and phage-treated groups (*p* < 0.01), confirming the potent bactericidal effect of P21 against B21 (Figure 3B). Similarly, when different phages (P49, P62, P70, P119, and PE17) were added one by one to different host bacteria during their logarithmic growth phases, bacterial growth was effectively inhibited. Turbidity decreased significantly in all five infection groups (*p* < 0.001), indicating that these five phages possess potent bactericidal activity (Figure 3B).

#### 3.7 Disruption of biofilm by phages application

**FIGURE 4:**
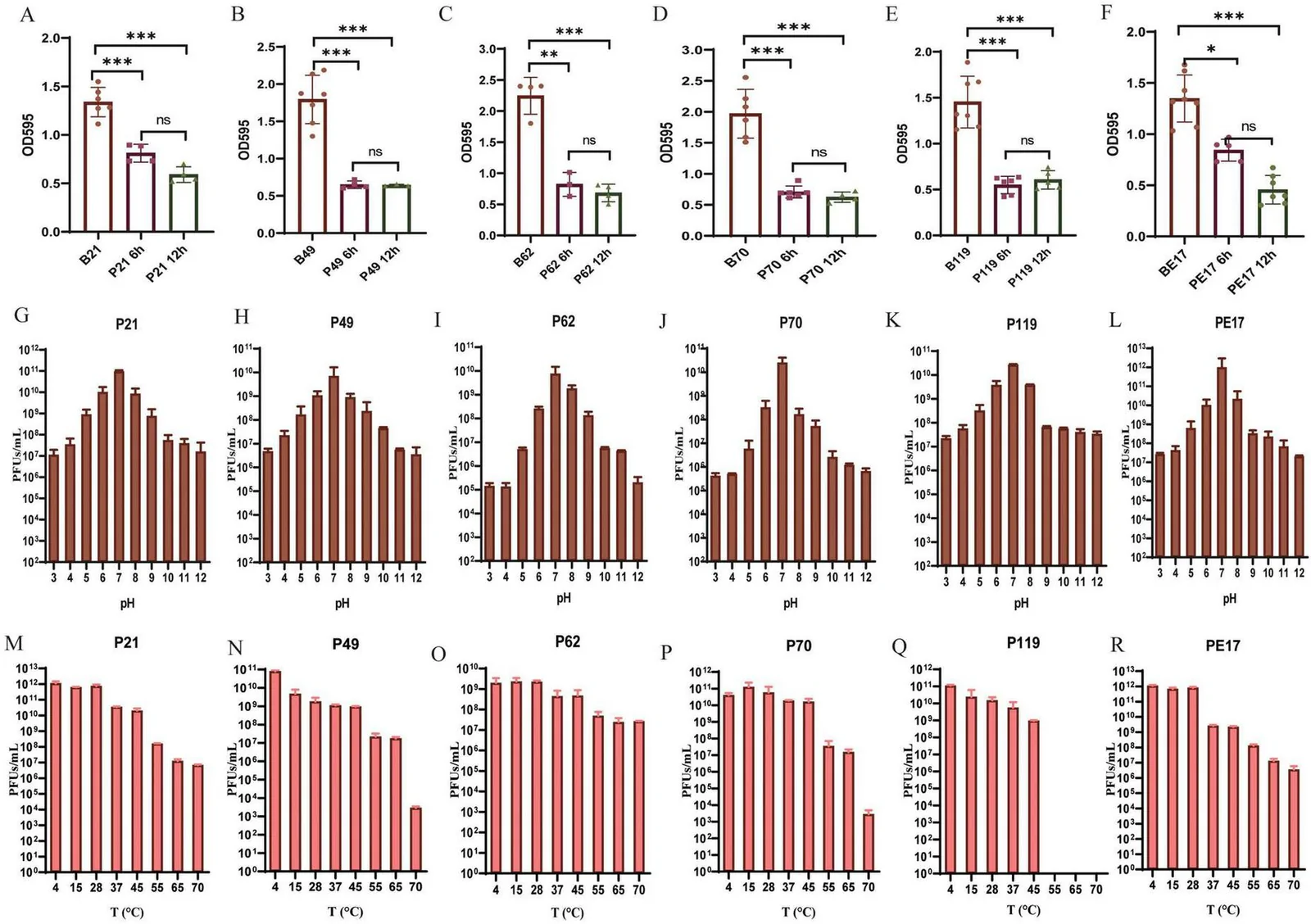
Disruption of biofilm by phages and stability assessment of phages. (A–F) The abilities of the six phages in degrading mature biofilms (*n* ≥ 5). (G–L) pH stability analysis, phage suspension was incubated at different pH values for 1 h. (M–R) Thermal stability analysis, phage suspension was incubated at different temperatures for 1 h; The results presented here are the mean values with SD indicated by error bars from three independent experiments. The stability of the six phages under different environmental conditions is evaluated, indicating their potential for practical applications. Values are expressed as means ± the SD. *p < 0.05; **p < 0.01, ***p < 0.001 (one-way ANOVA).

To evaluate the ability of bacteriophages to disrupt biofilm formation, mature biofilms were treated with phage suspensions (P21, P49, P62, P70, P119, and PE17) at their respective optimal MOIs for 6 and 12 h (Figures 4A–F). The results showed that all six phages significantly inhibited and disrupted E. coli biofilm formation at both time points, as evidenced by a marked reduction in OD_595_ values compared with the untreated control group (*P* < 0.001). This indicates that phage exposure effectively reduced biofilm biomass under the tested conditions. In addition, no statistically significant differences were observed between the 6-h and 12-h treatment groups for any of the phages (Figures 4A–F), suggesting that the antibiofilm activity of these phages was rapid and reached a plateau within the initial treatment period.

#### 3.8 Phage stability assessment

To evaluate the environmental robustness of the six phages, their thermal and pH stability were assessed over a broad range of temperatures (4–70°C) and pH values (3–12) (Figure 4). Overall, all six phages retained detectable infectivity across a relatively broad range of pH conditions, although their tolerance profiles differed among isolates.

The six phages showed distinct but generally broad pH tolerance profiles (Figures 4G–L). P49, P119, and PE17 retained detectable infectivity across almost the entire tested pH range (pH 3–12), although reductions in phage titers were observed under strongly acidic or alkaline conditions. P21 maintained detectable infectivity from pH 5 to 12, whereas P62 remained detectable over approximately pH 5–11. P70 showed a comparatively narrower pH tolerance range, with a marked reduction in infectivity under strongly acidic (pH ≤ 5) and alkaline conditions (pH ≥ 10). Overall, all six phages retained their highest or relatively high infectivity around neutral pH, although the magnitude of residual infectivity varied substantially among phages.

Thermal stability also differed markedly among the six phages (Figures 4M–R). P21 retained relatively high infectivity at temperatures between 4 and 28°C and remained detectable after exposure to 70°C, although its titer was substantially reduced. P62 and PE17 exhibited comparatively high thermal tolerance, retaining approximately 10^7^ PFU/mL after treatment at 70°C. P49 and P70 also retained detectable infectivity following exposure to 70°C, although their titers decreased to approximately 10^3^–10^4^ PFU/mL. In contrast, P119 was considerably more heat-sensitive, showing a pronounced loss of infectivity at 55°C and no detectable plaques after treatment at higher temperatures.

Taken together, the six phages exhibited phage-specific differences in resistance to pH and thermal stress. P49, P119, and PE17 showed broad pH tolerance, whereas P62 and PE17 exhibited relatively high thermal stability. The broad pH tolerance and considerable thermal resilience observed for several isolates may facilitate their further evaluation as candidates for phage-based antibacterial applications, although stability under physiologically relevant conditions and *in vivo* environments will require further validation.

#### 3.9 Genomic characteristics of the six phages

**FIGURE 5:**
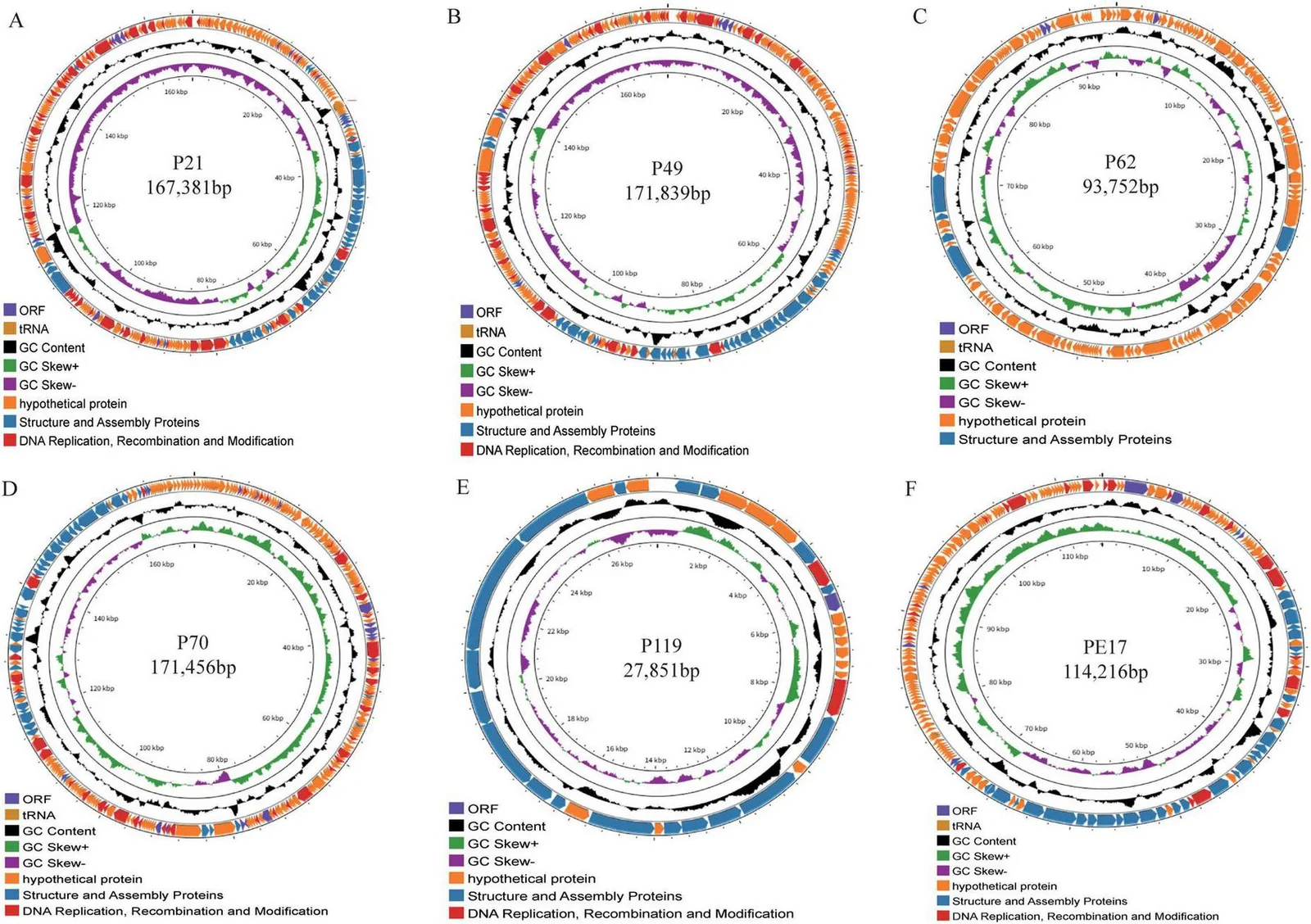
Genomic structural analysis of the six phages. Schematic representation of the genome structure of phage P21 **(A)**, P49 **(B)**, P62 **(C)**, P70 **(D)**, P119 **(E)**, and PE17 **(F)**. Genes of function are represented in dark blue. GC skew is indicated in orange for leading strand gene modules and in purple for lagging strand gene modules. The detailed genomic organization of the six phages is shown, with functional regions and gene orientations. Circular genome maps are shown for visualization purposes only.

To further investigate the genomic characteristics of the six phages (P21, P49, P62, P70, P119, and PE17), we analyzed their genome organization and the predicted functions of their encoded proteins (Table 1). Accordingly, the circular representations used in Figure 5 are genome-map representations for visualization and annotation. Genome assembly analysis suggested that the assembled contigs of P21, P49, and P70 could be circularized based on terminal overlaps, whereas no terminal overlaps were detected for P62, P119, and PE17, suggesting that these genomes represent linear assemblies.

The circularized genome assembly of phage P21 consists of a 167,381 bp double-stranded DNA sequence (GenBank accession: PZ583743), with a G + C content of 38.14% (Figure 5A and Table 1). The genome of P21 contains 10 tRNA genes. It encodes 266 open reading frames (ORFs) and the complete annotation of all predicted ORFs and functional assignments (Supplementary Table S3). Among these ORFs, 166 ORFs correspond to hypothetical proteins. Functional annotation predicted that 100 ORFs encode proteins with known functions. Specifically, 43 ORFs encode DNA replication, recombination and modification-related proteins, including exonuclease, DNA polymerase, and so on. 41 ORFs encode phage structure and assembly proteins, including tail proteins, baseplate wedge protein, major capsid protein, head protein, portal protein, terminase large subunit, terminase small subunit, and so on. 12 ORFs encode phage host takeover and regulation proteins, including tRNA ligase modifier, middle transcription regulatory protein MotA, and so on. Four ORFs encode core lysis-related proteins, including holin and endolysin.

The circularized assembled genome of phage P49 consists of a 171,839 bp double-stranded DNA sequence (GenBank accession: PZ583739), with a G + C content of 40.16% (Figure 5B and Table 1). The genome of P49 contains 2 genes encoding tRNA-Met and tRNA-Arg. This genome encodes 294 ORFs (Supplementary Table S4). There are 205 hypothetical proteins. Of these ORFs, 38 encode DNA replication, recombination and modification related proteins, such as endonuclease, recombination endonuclease, DNA polymerase, ATP-dependent DNA ligase, putative DNA helicase, and DNA primase. 36 ORFs are predicted to encode proteins with structure and assembly proteins, such as internal protein, tail protein, structural protein, major capsid protein, head protein, portal protein, terminase large subunit. Four ORFs encode cell lysis-associated proteins, which include lysis inhibition accessory protein, antiholin, endolysin, and holin.

Phage P62 has an assembled linear double-stranded DNA sequence of 93,752 bp in length (GenBank accession: PZ583741), with a G + C content of 45% (Figure 5C and Table 1). Based on the available genomic evidence, P62 was assigned to *Caudoviricetes incertae sedis* (Figure 5C and Table 1). The genome of P62 contains a gene encoding tRNA-Arg. This genome encodes 153 ORFs (Supplementary Table S5). There are 149 hypothetical proteins. Three ORFs encode lysis-associated proteins, including endolysin, SAR-endolysin, and U-spanin. One ORF encode tail tip protein L, classified as a phage structural protein.

The circularized genome of phage P70 consists of a 171,456 bp double-stranded DNA sequence (GenBank accession: PZ583742), with a G + C content of 41% (Figure 5D and Table 1). The genome of P70 contains 2 genes encoding tRNA-Met and tRNA-Arg. It encodes 296 open reading frames (ORFs) and the complete annotation of all predicted ORFs and functional assignments (Supplementary Table S6). Among these ORFs, 207 ORFs correspond to hypothetical proteins. Functional annotation predicted that 89 ORFs encode proteins with known functions. Specifically, 38 ORFs encode DNA replication, recombination and Modification related proteins, such as exonuclease, DNA polymerase, recombination and repair protein, DNA primase, ribonuclease H, thymidylate synthase. 36 ORFs encode phage structure and assembly proteins, such as baseplate protein, major capsid protein, head protein, portal protein, tail protein, terminase large subunit, and terminase small subunit. 11 ORFs encode phage host takeover and regulation proteins, including tRNA ligase modifier, middle transcription regulatory protein MotA, and so on. Four ORFs encode core lysis-related proteins, including lysis inhibition accessory protein, antiholin, endolysin, and holin.

The current assembly of phage P119 consists of a 27,851 bp linear double-stranded DNA sequence (GenBank accession: PZ583740), with a G + C content of 40%. No tRNA genes were detected (Figure 5E and Table 1). Phage P119 genome encodes 35 ORFs (Supplementary Table S7). There are 16 hypothetical proteins. Two ORFs encode DNA replication, recombination and modification related proteins, including deoxynucleotide monophosphate kinase and RNA ligase. 7 ORFs encode structure and assembly proteins, including baseplate wedge protein, tail tube protein, tail fiber protein, major capsid protein, prohead assembly protein gp68, portal protein, and baseplate puncturing device.

The assembled genome of phage PE17 consists of a 114,216 bp linear double-stranded DNA sequence (GenBank accession: PZ583738), with a G + C content of 38.57% (Figure 5F and Table 1). The genome of PE17 contains 2 genes encoding tRNA-Met and tRNA-Arg. This genome encodes 195 ORFs (Supplementary Table S8). There are 137 hypothetical proteins and 58 putative annotated proteins. 20 ORFs encode DNA replication, recombination and modification related proteins, such as endonuclease, recombination endonuclease, DNA polymerase, ATP-dependent DNA ligase, putative DNA helicase, and DNA primase. 33 ORFs are predicted to encode structure and assembly proteins, such as tail protein, structural protein, major capsid protein, head protein, portal protein, and terminase large subunit. Three ORFs encode cell lysis-associated proteins, which include lysis inhibition accessory protein, antiholin, and endolysin. Two ORFs encode phage host takeover and regulation proteins, including Valyl-tRNA ligase modifier and RNA polymerase sigma factor.

To evaluate the presence of genomic features potentially associated with safety concerns, the genomes and predicted proteins of the six phages were screened against antibiotic resistance and virulence databases. Searches against CARD and ResFinder identified no acquired antibiotic resistance determinants, and no known virulence-factor genes were detected. In addition, no integrase or recombinase genes commonly associated with lysogeny were identified in any of the six genomes. While this absence is consistent with a lytic lifestyle, it does not alone constitute definitive proof. Therefore, based on the combined evidence from genome annotation, plaque morphology, and infection kinetics, we described the six phages as lytic (Table 1).

#### 3.10 Phylogenetic analysis of the six phages

To classify and identify phages P21, P49, P62, P70, P119, and PE17, this study employed a comprehensive approach combining BLASTn sequence similarity analysis from the NCBI database, in combination with the proteomic tree constructed by VipTree, a whole-genome phylogenetic tree based on Genome BLAST Distance Phylogeny (GBDP) method, and the OrthoANI average nucleotide identity heatmap. The integrated analyses supported the assignment of P21 to the genus *Tequatrovirus* and P49, P70, P119, and PE17 to the genus *Gaprivervirus*, all within the family *Straboviridae*. In contrast, P62 could not be confidently assigned to a currently recognized genus and was therefore conservatively retained as *Caudoviricetes incertae sedis* (Table 1).

**FIGURE 6:**
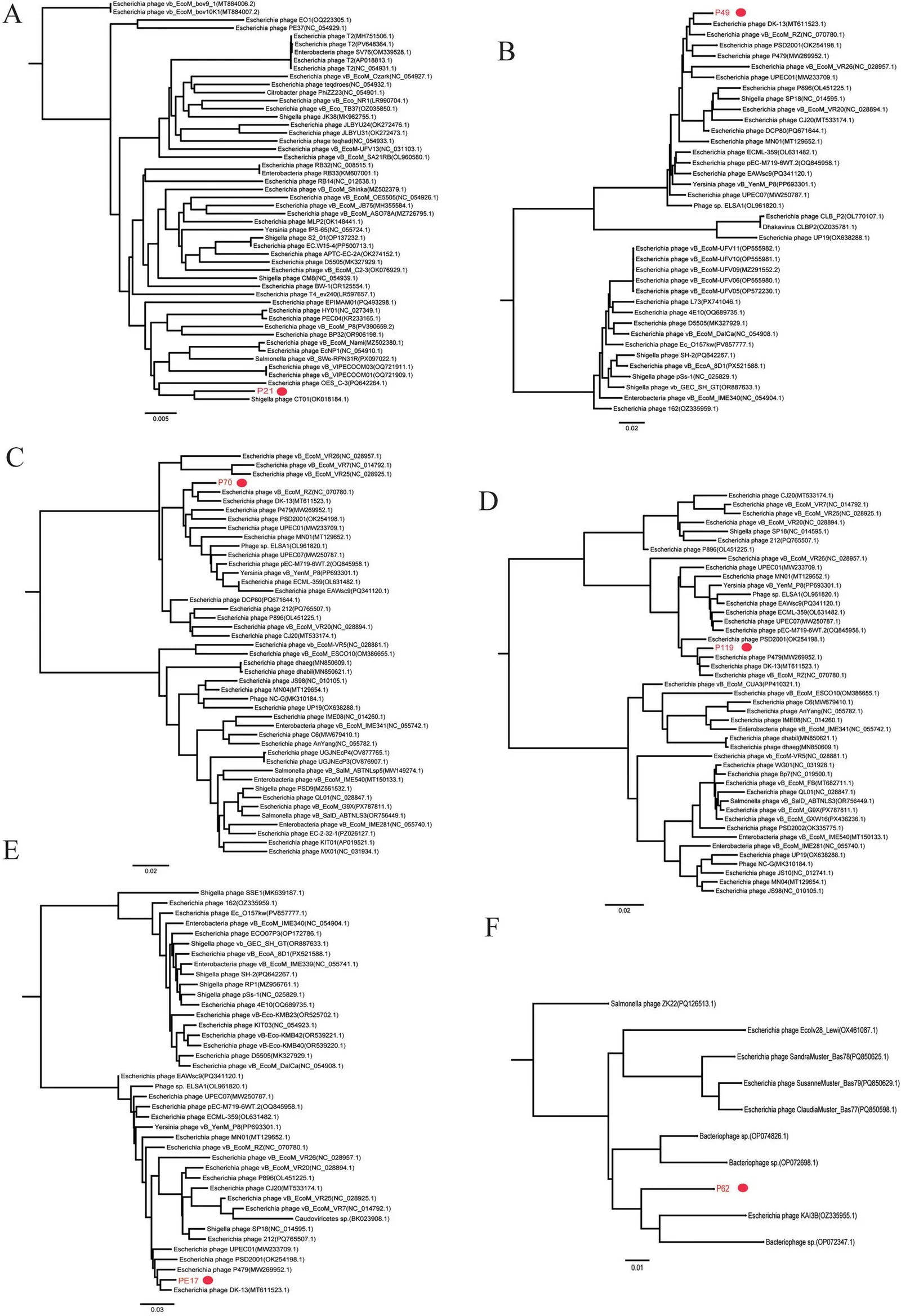
Whole-genome BLASTn-based phylogenetic tree showing the relationship of the six phages to the top BLASTn-matched Escherichia phages. **(A)** P21, **(B)** P49, **(C)** P70, **(D)** P119, **(E)** PE17, **(F)** P62.

**FIGURE 7:**
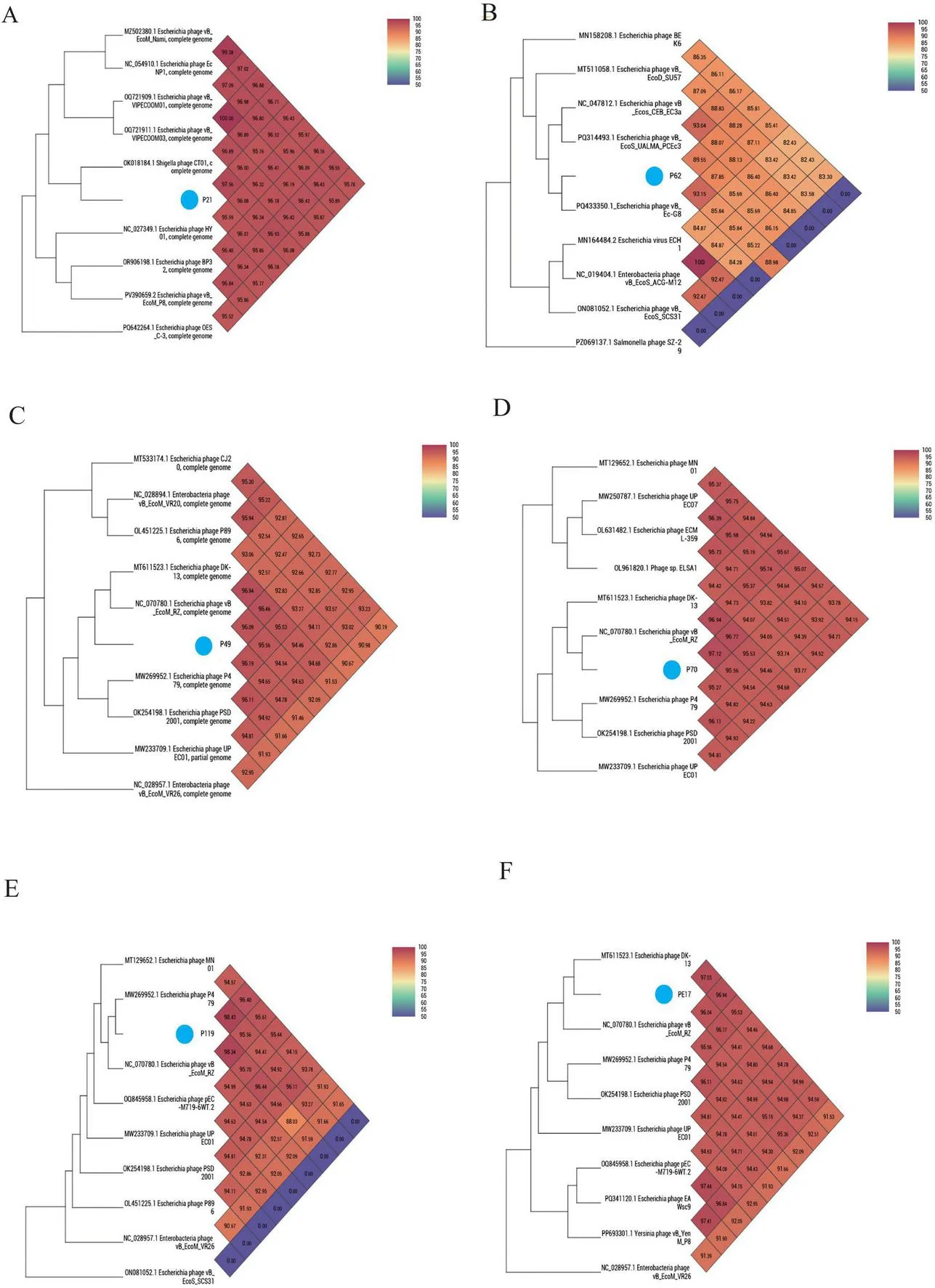
Phylogenetic and ANI analysis of phages related to the six phages. **(A–F)** ANI analysis and clustering of phage genomes for P21 **(A)**, P62 **(B)**, P49 **(C)**, P70 **(D)**, P119 **(E)**, and PE17 **(F)** against related species. The ANI was constructed using the OrthoANI tool based on complete genomic sequences. Heatmaps visually represent the genomic similarity among the compared phages. These heatmaps confirm the genomic divergence of the novel phages, reinforcing their classification as new species and potential new genera.

**FIGURE 8:**
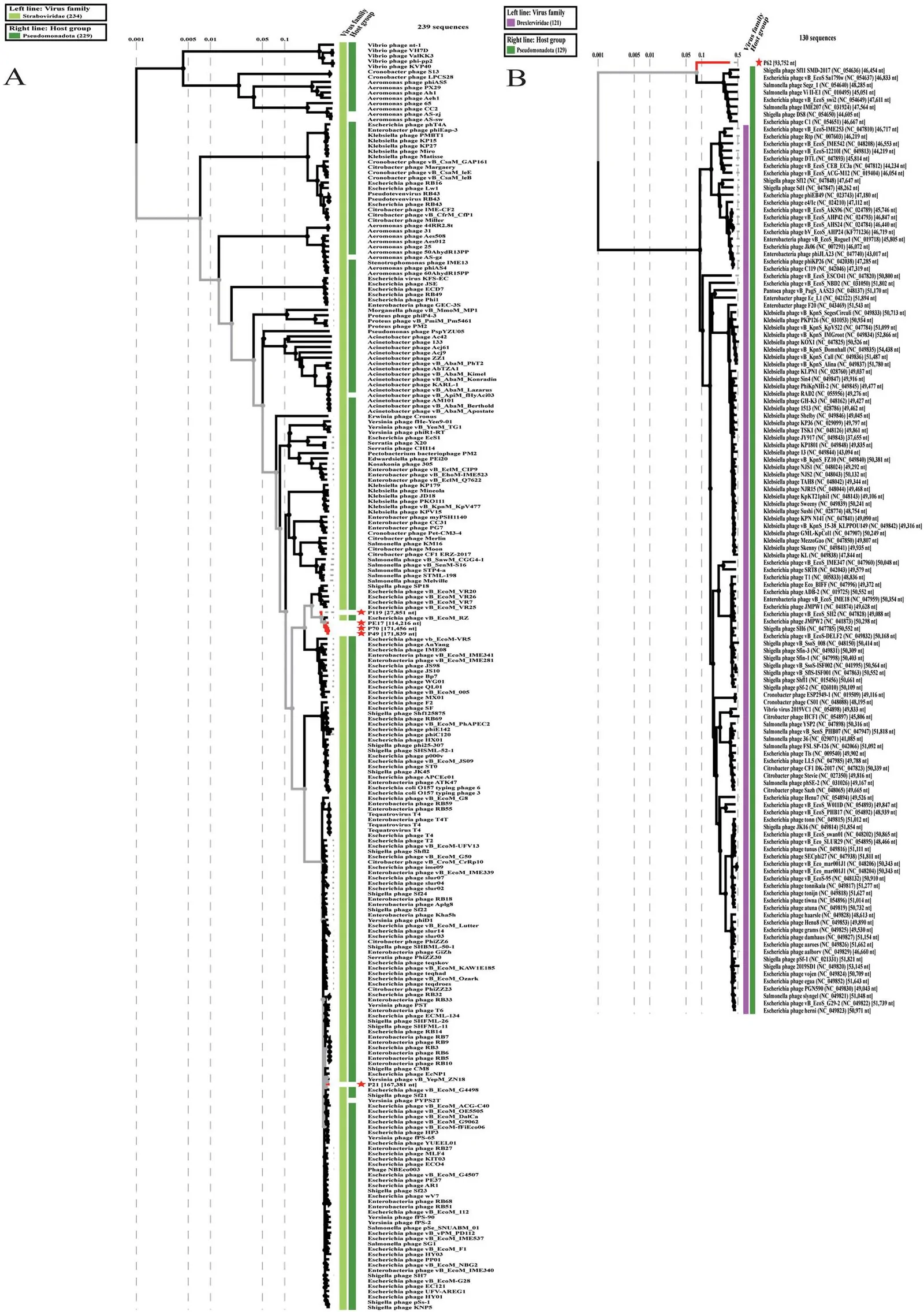
Whole-proteome (genome-wide) similarity tree generated using ViPTree (v3.3), showing the placement of the six pahges among related phages. Phage P21, P62, P49, P70, P119, and PE17 is highlighted and clusters nearest *Escherichia* phages, with marked with red five-pointed stars. Proteomic tree of phages **(A)** P21, P62, P49, P70, P119, and PE17, **(B)** constructed using VipTree. The tree includes all phages that exhibited a similarity score above zero. Phages marked with light green squares belong to the *Straboviridae* family, while phages marked with green squares have host bacteria classified under the Pseudomonadota phylum, and phages marked with purple squares belong to *Drexlerviridae* families. The branch lengths shown in the figure represent the accumulated evolutionary distance along that branch, measured in the number of substitutions per site.

A comparative analysis was performed for P21 using NCBI BLASTn, whole-genome phylogenetic analysis, ViPTree proteomic analysis, and ANI analysis. NCBI BLASTn initially identified several closely related phage genomes, among which *Shigella* phage CT01 (OK018184.1) showed the closest phylogenetic relationship to P21. In the whole-genome phylogenetic tree, P21 and CT01 clustered within the same branch (Figure 6A). To further evaluate the genomic relationships of P21, the nine closest genomes identified by the BLASTn search were selected for ANI analysis. P21 showed an ANI of approximately 97.0% with CT01, 95.59% with *Escherichia* phage HY01 (NC_027349.1), and 96.52% with *Escherichia* phage EcNP1(NC_054910.1) (Figure 7A). These results demonstrated a high level of genome-wide similarity between P21 and several closely related phages. The ViPTree proteomic analysis further placed P21 within the lineage containing *Tequatrovirus*-associated phages (Figure 8A).

The whole-genome phylogenetic tree results show that P49 is in the same major evolutionary branch as *Escherichia* phage DK-13 (MT611523.1), phage vB_EcoM_RZ (NC_070780.1), indicating a close phylogenetic relationship (Figure 6B). The results showed that the values of ANI 96.46 and 96.09% (Figure 7C) classified it under the genus *Gaprivervirus*.

A comparative analysis using BLASTn, ANI, whole-genome phylogeny, and ViPTree further supported the P70, P119, and PE17 within the genus *Gaprivervirus* (Table 1). The P70 closest match was *Escherichia* phage vB_EcoM_RZ(NC_070780.1) (ANI 97.12%) and *Escherichia* phage DK-13(MT611523.1) (ANI 96.77%) (Figures 6C, 7D). The P119 closest match was *Escherichia* phage PSD2001(OK254198.1) (ANI 94.66%), phage P479(MW269952.1) (ANI 98.43%) and phage vB_EcoM_RZ (NC_070780.1) (ANI 98.34%) (Figure 6D, 7E). The PE17 closest match was *Escherichia* phage P479(MW269952.1) (ANI 96.17%), phage DK-13 (MT611523.1) (ANI 97.03%) and *Escherichia* phage vB_EcoM_VR26 (NC_028957.1) (ANI 92.51%) (Figures 6E, 7F). These supporting placement within *Gaprivervirus* while suggesting a distinct species-level lineage under commonly used demarcation criteria (Figures 6, 7). Using the whole-genome tBLASTx method, the proteomic phylogenetic tree generated by VipTree indicated that P49, P70, P119, and PE17 clustered within the same large branch as *Escherichia* phage vB_EcoM_VR25, *Escherichia* phage vB_EcoM_RZ, and *Escherichia* phage vb_EcoM-VR5 (Figure 8A).

BLASTn the phage P62 whole-genome phylogenetic analysis and ANI revealed that the ANI of P62 with *Escherichia* phage vB_Ec-G8 was 93.15%, and its genomic ANI with other *Escherichia* phages (BEK6 and ECH1) was below 90% (Figures 6F, 7B). As shown in the VipTree-based proteomic tree (Figure 8B), P62 formed a monophyletic cluster with *Escherichia* phage vB_EcoS_Sa179lw (NC_054637) and *Shigella* phage Sf11_SMD-2017 (NC_054636). According to the phage classification criteria proposed by Dann Turner, ANI similarity ≥ 95.00% defines the same species, ≥ 70.00% defines the same genus (Turner et al., 2021). The supporting placement P62 within *Caudoviricetes incertae sedis*, while suggesting a distinct species-level lineage under commonly used demarcation criteria. Moreover, there was no genomic similarity between *Salmonella* phage SZ-29 (PZ069137.1). Thus, from an evolutionary and taxonomic perspective, P62 represents a novel species.

To complement the genome-wide proteomic analysis, we constructed a maximum-likelihood phylogeny using the terminase large subunit, a widely used, conserved marker for tailed-phage classification, and the major capsid protein (mcp), phage Endolysin (Supplementary Figure S2). Then, further opted for sequence alignment through BLASTp, followed by visualizing the phylogenetic relationships with closely related proteins using MEGA. As observed in Supplementary Figure S2A–I, orthologous phages P21, P49, P70, PE17, P119 mcp, and TerL proteins were grouped and clustered with those of other *Escherichia* phages. Since no MCP or TerL homologs were annotated in the P62 genome, we constructed a phylogenetic tree based on its putative endolysin. The resulting tree (Supplementary Figure S2J) placed the P62 endolysin within a clade containing *Escherichia* phage endolysins and several *Klebsiella* phages, indicating its endolysin has a closer evolutionary affinity to *Klebsiella*-associated phages.

**FIGURE 9:**
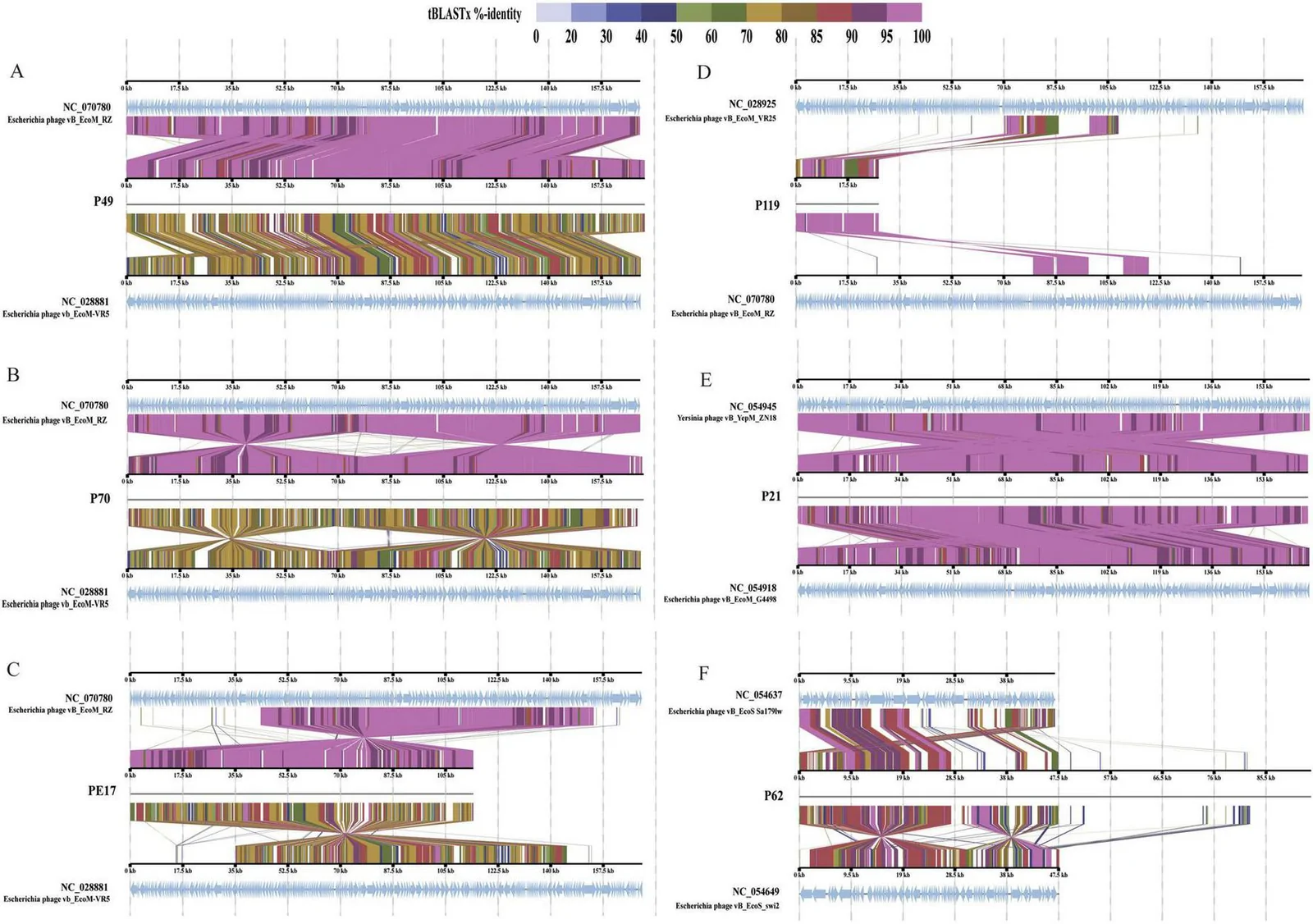
Collinearity analysis of the six phage genomes. **(A–C)** Genome collinearity analysis of phage P49, P70, PE17 with *Escherichia* phage vB_EcoM_RZ and phage vb_EcoM-VR5. **(D)** Genome collinearity analysis of phage P119 with *Escherichia* phage vB_EcoM_VR25 and phage vB_EcoM_RZ. **(E)** Genome collinearity analysis of phage P21 with *Escherichia* phage vB_EcoM_G4498 and *Yersinia* phage vB_YepM_ZN18. **(F)** Genome collinearity analysis of phage P62 with *Escherichia* phage vB_EcoS Sa179lw and phage vB_EcoS_swi2. The color gradient from light purple to light reddish-purple indicates the gradual increase in BLASTn similarity from 0.00 to 100.00%. Reverse connecting lines represent genomic segment rearrangements. Arrow directions indicate the orientation of genomic sequences or genes, illustrating the relationship between different regions of the genome. The square diagram on the left corresponds to the arrows and lines in the collinearity plot on the right, showing the comparison and similarity between different genomes. The collinearity of phage genomes is displayed, showing sequence alignment and genomic rearrangements for comparative analysis.

To further examine genome organization and conservation, a genome collinearity analysis was performed between the six phages and their closest phylogenetic relatives’phages (Figure 9). The results revealed a high degree of genomic similarity across most regions (over 70.00% similarity, Figure 9), indicating a high level of evolutionary conservation among these phages. Notably, some regions of phage P49 exhibited up to 90.00% similarity with *Escherichia* phage vB_EcoM_RZ (Figure 9A). Phage P70 and PE17 exhibited about to 85% similarity with vB_EcoM_RZ (Figures 9B, C). In contrast, the genome collinearity analysis between phage P49, P70, PE17, and vb_EcoM-VR5 demonstrated lower overall similarity, with several regions showing similarity below 80.00%, and multiple extended regions with no matching sequences (Figures 9A–C). Phage P119 exhibited about to 85% similarity with *Escherichia* phage vB_EcoM_VR25 and phage vB_EcoM_RZ (Figure 9D). Phage P21 exhibited up to 90.00% similarity with *Escherichia* phage vB_EcoM_G4498 and *Yersinia* phage vB_YepM_ZN18 (Figure 9E). Phage P62 exhibited about to 85% similarity with *Escherichia* phage vB_EcoS Sa179lw and phage vB_EcoS_swi2 (Figure 9F). Specifically, P49, P21, P119 several gene regions, including structure and assembly proteins, corresponding matches. These genes encode proteins of known function, such as tail protein, head protein, and baseplate wedge protein. DNA replication, recombination and modification gene regions of the PE17, including structure and assembly proteins, lacked corresponding matches. These genes encode proteins of unknown function, endonucleases. All of this, the six phages exhibit high collinearity with related phages across the essential modules (structure and assembly proteins and lysis proteins), whereas several regions diverge, including segments enriched for hypothetical proteins (Figure 9). These differences support genomic divergence within the different phage lineages and highlight candidate regions for future functional investigation (Zhang et al., 2023; Zhou et al., 2015).

### 4 Discussion

In this study, six lytic bacteriophages infecting MDR *E. coli* were isolated from hospital wastewater and comprehensively characterized through phenotypic, physicochemical, morphological, and genomic analyses. The phages differed in infection kinetics, MOI, physicochemical stability, genome organization, and taxonomic position. Based on whole-genome comparative and phylogenetic analyses, P21 was assigned to the genus *Tequatrovirus*, whereas P49, P70, P119, and PE17 were classified within the genus *Gaprivervirus*. P62 was assigned to a potentially novel lineage within *Caudoviricetes* based on genome-based taxonomic analysis. In addition, no known antibiotic resistance genes, virulence associated genes, or lysogeny-related modules were identified in any genome. These findings expand the available diversity of MDR *E. coli* infecting phages and provide additional insights into the biological characteristics that may influence phage selection.

The successful isolation of six phages from hospital wastewater highlights the value of this environment as a source of bacteriophages associated with clinically relevant bacterial hosts. Hospital wastewater contains diverse bacterial populations and their associated phages and is continuously influenced by antimicrobial exposure, bacterial shedding, and horizontal gene transfer, which may promote phage-host interactions and phage diversity (Barrero-Canosa et al., 2023; Huang et al., 2025b). The MDR phenotype of the host strain further emphasizes the clinical relevance of these isolates, as resistance to multiple antibiotic classes can substantially limit conventional treatment options. The successful recovery of multiple phages from the same wastewater source therefore highlights the potential of hospital wastewater for discovering phages targeting clinically relevant antibiotic-resistant *E. coli* (Imamovic et al., 2010). Broader sampling strategies and culture-independent approaches will be necessary to better understand the diversity and ecological distribution of *E. coli* infecting phages in such environments.

The six phages showed efficient adsorption and relatively rapid infection kinetics, although their infection profiles differed among isolates. More than 90% of the phage particles were adsorbed within the tested period, indicating efficient attachment to the host under the experimental conditions. The latent periods and subsequent infection dynamics varied among phages, with PE17 showing particularly rapid infection, whereas P49, P70, and P119 exhibited moderate latent periods of approximately 15–20 min. These values are broadly comparable to those reported for other lytic *E. coli* phages (Alrahimi et al., 2026; Niaz et al., 2025; Shyrokova et al., 2025). These phages may encode efficient lysis-associated proteins that facilitate rapid host takeover and progeny release (Loessner, 2005).

The optimal MOI also varied among the six phages, ranging from 0.1 to 10. This variation indicates that the effective phage-to-host ratio is dependent on the specific phage-host combination and experimental conditions rather than representing a universal characteristic of lytic phages (Huang et al., 2025b; Yao et al., 2023). The MOI values determined in this study represent conditions that achieved effective bacterial inhibition *in vitro* rather than universal indicators of phage fitness or therapeutic dosage. Factors such as bacterial density, growth state, receptor availability, and environmental conditions can influence phage infection outcomes.

Physicochemical characterization revealed that the six phages maintained infectivity across broad ranges of pH and temperature, although individual phages showed different tolerance profiles. This represents a relatively broad stability range compared with many characterized bacteriophages, which generally show maximal stability near neutral pH and progressively reduced infectivity under strongly acidic or alkaline conditions (Huang et al., 2025b; Wang et al., 2024; Yao et al., 2023). The ability of the six isolates to remain infectious across such a wide pH range is therefore an important phenotypic feature and may contribute to their persistence under fluctuating environmental conditions. Importantly, all phages retained infectivity under acidic conditions (pH < 4), although with variable reductions in titer, indicating potential resilience in fluctuating physiological environments. However, given that most infection sites (e.g., blood, cerebrospinal fluid, and urine) maintain near-neutral pH (7.2–7.4), this stability profiles are compatible with potential systemic or localized therapeutic applications (Chen et al., 2025; Dąbrowska, 2019; Jędrusiak et al., 2023).

The thermal stability profiles further revealed substantial phenotypic variation among the isolates. P21, P49, P62, P70, and PE17 retained considerable infectivity at elevated temperatures, including detectable infectivity at 65°C, whereas P119 showed a more pronounced reduction in titer above 55°C. The relatively high thermal tolerance of several isolates is consistent with the possibility that their virion structures remain stable under conditions that are unfavorable to many phages, which is higher than that reported for many comparable bacteriophages (Chen et al., 2025; Khalifa and Omar, 2025; Niaz et al., 2025). In contrast, the greater sensitivity of P119 to elevated temperatures suggests that its preservation may require more controlled conditions, particularly refrigerated storage. Although genome comparisons revealed differences in genome size, predicted proteins, and taxonomic classification, the molecular determinants responsible for pH and thermal tolerance remain unclear. Phage stability is influenced by the structural integrity and physicochemical properties of the capsid, portal, tail, and other virion-associated proteins (Poranen et al., 2002). Physiological environments contain proteins, salts, bile components, nucleases, immune factors, and other interacting molecules that can affect virion integrity and infectivity (Jończyk-Matysiak et al., 2019). Many predicted proteins in environmental phage genomes remain functionally uncharacterized, limiting the ability to associate specific genes with observed phenotypes.

The six phages showed substantial genomic and phylogenetic diversity. Their genome sizes, accompanied by marked differences in ORF number, tRNA content, GC content, and predicted functional repertoires (Table 1). The recovery of genetically distinct phages from the same ecological niche suggests that wastewater associated phage communities contain evolutionary diversity. P62, which occupied an unresolved position within Caudoviricetes, further illustrates the limitations of current phage reference databases and the continued discovery of previously underrepresented viral lineages. In addition, a substantial proportion of predicted ORFs remained annotated as hypothetical proteins, highlighting the incomplete understanding of phage gene functions and the complexity of phage-host evolutionary relationships (Del Arco et al., 2024).

The genomic characteristics of the six isolates also provide initial information regarding their suitability for further investigation. Screening against available databases did not identify known antibiotic resistance determinants, virulence-associated genes, or lysogeny-related genes, supporting their classification as potentially suitable lytic phage candidates for additional evaluation (Hyman, 2019; Strathdee et al., 2023). However, the absence of detectable lysogeny-associated genes does not by itself establish an obligately lytic lifestyle, and genomic screening alone cannot establish complete biological safety. The absence of detectable genes in these databases does not by itself establish phage safety or definitively exclude lysogenic potential. Further experimental validation would be valuable for confirming the absence of lysogen formation. The presence of uncharacterized proteins, potential genome instability, contamination during phage preparation, and host-related responses require consideration in subsequent studies (Hyman, 2019; Niazi, 2025).

All six phages significantly reduced *E. coli* biofilm biomass under the tested conditions. The lack of significant differences between 6 and 12 h treatments indicate that most measurable biofilm reduction occurred during the early stage of exposure. Several mechanisms may contribute to phage mediated biofilm reduction, including infection of biofilm-associated bacterial cells and interactions between phage particles and extracellular biofilm components (Ferriol-González and Domingo-Calap, 2020). However, because this study did not directly investigate phage penetration, extracellular matrix degradation, or specific phage encoded biofilm associated functions, the molecular basis of the observed antibiofilm activity remains to be determined.

The six phages exhibited substantial diversity in adsorption kinetics, infection dynamics, physicochemical stability, genome organization, and taxonomic affiliation. Such diversity may provide useful information for phage selection, as phages with distinct host interactions and infection characteristics may exert different selective pressures on bacterial populations (Cook and Hynes, 2025; Naureen et al., 2020). However, phage diversity alone does not guarantee improved cocktail performance, and factors including host receptor usage, adsorption efficiency, replication characteristics, and resistance profiles should be considered during phage combination design (Maffei et al., 2021; Niazi, 2025).Therefore, further experimental validation is required to determine whether specific combinations provide advantages over individual phages (Aranaga et al., 2022; Johri et al., 2023; Kaneko et al., 2026; Kim et al., 2025; Kortright et al., 2019; Rotman et al., 2024; Unnikrishnan et al., 2023; Zhao et al., 2024).

Several limitations should be considered when interpreting these findings. First, the phages were isolated from a single wastewater source and evaluated against a limited number of bacterial strains; therefore, their broader host range and ecological distribution remain unknown. Second, the phenotypic assays were conducted under controlled laboratory conditions and may not fully represent phage behavior in complex environments. Third, although genomic screening did not identify known undesirable genetic elements, the functional roles of many predicted proteins remain unresolved. Further studies focusing on host-range determination, resistance evolution, phage interactions, and validation in biologically relevant models will be required to better understand the potential applications of these isolates. Overall, this study provides a diverse set of MDR *E. coli* infecting phages and establishes a foundation for further investigation of phage diversity, biology, and selection strategies.

### 5 Conclusion

This study expands the known diversity of *Escherichia* bacteriophages and identifies six novel lytic phages with favorable biological properties against MDR *E. coli*. Their stability, rapid infection kinetics, and taxonomic novelty highlight their potential as candidates for future therapeutic development. Notably, these findings strongly advocate for the continued development of phage therapy, especially through the design of rationally formulated phage cocktails, as a promising strategy to combat MDR bacterial infections.

## Data Availability

The data analyzed in this study is available in the NCBI Genbank (https://www.ncbi.nlm.nih.gov/genbank) under accession numbers PZ583738-PZ583743.

## Footnotes

1
https://viralzone.expasy.org/

2
http://www.genome.jp/kegg/

3
https://www.ncbi.nlm.nih.gov/COG/

4
http://www.mgc.ac.cn/cgi-bin/VFs/v5/main.cgi?func=VFanalyzer

5
https://card.mcmaster.ca/analyze

6
https://lowelab.ucsc.edu/tRNAscan-SE/

7
https://phage.ee.cityu.edu.hk/
