# Supplementary material for: Genomic and functional characterization of six novel phages targeting multidrug-resistant Escherichia coli: promising candidates for clinical phage therapy
Source: Front Microbiol. 2026 Sep 10;17:1921205. doi: 10.3389/fmicb.2026.1921205 (PMC13602300; doi:10.3389/fmicb.2026.1921205)
Supplement: Supplementary file 1 [file Supplementary_file_1.docx]

**Supplementary material**

Fig.S1 Lysis curves for the six phages

Fig.S2 Phylogenetic analysis of the three signature proteins of the six phage and top matched BLASTp of other Phages using MEGA.

(A-E) Phage P21, P49, P70, PE17, P119 major capsid protein (mcp) phylogenetic tree.

(F-I) Phage P21, P49, P70, PE17 Terminase large subunit (TerL) phylogenetic tree.

(J) Phage P62 Endolysin tree.
